# Cholera Toxin-Mediated Targeting of Botulinum Neurotoxin Activity to Pain-Associated Sensory Neurons

**DOI:** 10.3390/toxins18040174

**Published:** 2026-04-03

**Authors:** Eve Corrie, Rebecca Bresnahan, Ciara Doran, Charlotte Leese, Matthew R. Balmforth, Anna Andreou, Aisha Zhantleuova, Elizabeth P. Seward, Michael E. Webb, W. Bruce Turnbull, Bazbek Davletov

**Affiliations:** 1 School of Biosciences, University of Sheffield, Sheffield S10 2TN, UK; eve.corrie@md.catapult.org.uk (E.C.); rebecca.bresnahan@liverpool.ac.uk (R.B.); c.doran@sheffield.ac.uk (C.D.); c.leese@sheffield.ac.uk (C.L.); e.p.seward@sheffield.ac.uk (E.P.S.); 2 School of Chemistry and Astbury Centre for Structural Molecular Biology, University of Leeds, Leeds LS2 9JT, UK; mattrbalmforth@hotmail.com (M.R.B.); m.e.webb@leeds.ac.uk (M.E.W.); w.b.turnbull@leeds.ac.uk (W.B.T.); 3 Headache Research, Wolfson Sensory, Pain and Regeneration Centre, King’s College London, London SE1 1UL, UK; anna.andreou@kcl.ac.uk; 4 Department of Biophysics, Biomedicine and Neuroscience, Al-Farabi Kazakh National University, Almaty A15E3C7, Kazakhstan

**Keywords:** botulinum neurotoxin, cholera toxin, neuropathic pain, chimeric toxin, calcitonin gene-related peptide (CGRP), analgesic

## Abstract

Botulinum neurotoxin injections are used off-label to treat chronic pain, but their efficacy is limited and paralytic effects restrict clinical utility in these applications. Here, we investigated whether combining the light chain and translocation domains of botulinum neurotoxin A (BoNT/A) with the GM1-binding B subunit of cholera toxin would be beneficial in silencing pain-associated sensory neurons. Chimeric ChoBot was assembled via a coiled-coil linking technology and was shown to retain the enzymatic activity of BoNT/A in vitro and in vivo. In cultured dorsal root ganglion neurons, ChoBot cleaved SNAP25 in a calcitonin gene-related peptide (CGRP)-rich subpopulation of sensory neurons, resulting in marked inhibition of CGRP release. ChoBot had a lesser effect on the compound muscle action potentials of the rat gastrocnemius muscle than BoNT/A following subcutaneous injections. In rat models of pain, including chemotherapy-induced peripheral neuropathy, intraplantar administration of ChoBot significantly attenuated mechanical allodynia. Immunohistochemical analysis confirmed SNAP25 cleavage in NF200- and CGRP-expressing sensory fibres in the epidermis following a single injection. ChoBot also mediated SNAP25 cleavage in human neuroblastoma cells in culture. Together, these findings indicate that ChoBot enables a silencing of pain-associated sensory pathways, providing a new strategy for the development of new long-lasting analgesics for chronic pain.

## 1. Introduction

Neuropathic pain is estimated to affect up to 10% of the general population and arises from damage or disease of the somatosensory nervous system [1,2]. This form of chronic pain serves no protective advantage to the body and is difficult to manage with current therapies such as opioids, anticonvulsants/calcium channel α2-δ ligands and antidepressants—all requiring daily intakes [3,4]. There is a clear need to develop safe, long-lasting analgesics that can specifically treat chronic neuropathic pain.

Botulinum neurotoxins (BoNTs), which are capable of neuronal silencing lasting many months, are produced by several strains of the *Clostridium* bacteria [5]. Presently, BoNTs are characterised into at least eight different major serotype groups, BoNT/A to BoNT/H [6]. The shared mechanism of these different BoNTs is that they are all proteases that cleave soluble N-ethylmaleimide-sensitive factor attachment protein receptor (SNARE) proteins at the presynaptic terminals of neurons [7]. The SNARE proteins are found on both the vesicle membrane and the cell membrane and are known to form a tetra-helical complex that facilitates membrane fusion, leading to the secretion of neurotransmitters and neuropeptides from neurons [8]. BoNTs truncate specific SNARE proteins, impairing SNARE complex formation and thereby preventing vesicle fusion and exocytosis, leading to muscle paralysis and death.

The 150 kDa BoNTs are composed of three domains: a light chain (Lc) at the N-terminal end and a heavy chain consisting of translocation domain (Td) and receptor-binding domain at the C-terminus [9]. Native BoNTs have specificity for peripheral cholinergic nerve terminals at the neuromuscular junction due to the receptor binding domain interacting with two receptors, typically a ganglioside and a vesicle protein receptor, allowing the neurotoxin to be taken up into motor nerve terminals by endocytosis [10,11]. The light chain is an active zinc endoprotease that proteolyses a specific peptide bond on a specific SNARE protein, which differs between BoNT serotypes [12]. This cleavage at the neuromuscular junction prevents acetylcholine release, leading to flaccid paralysis [13]. While BoNTs are used widely to treat muscular conditions, they were also shown to have analgesic potential since they can also act on sensory neurons [14,15,16,17,18].

BoNT type A (BoNT/A) exerts its long-lasting effects by cleaving off only nine amino acids from the SNARE protein synaptosomal-associated protein 25 (SNAP25) [14]. The slightly truncated SNAP25 forms a deficient SNARE complex incapable of membrane fusion. The BoNT/A serotype has been most extensively studied for its analgesic properties, in part because of its longest duration of action compared to other serotypes and has been approved by the FDA to treat chronic migraine [15]. BoNT/A also shows efficacy for the treatment of neuropathic pain conditions, but the proportion of patients responding and the degree of pain relief is relatively low, a common trait of the current treatments for neuropathies [16,17,18]. One proposed mechanism of BoNT/A analgesia is the inhibition of neurotransmitter release from primary afferent nociceptors onto the spinal cord [19,20]. BoNT/A appears to inhibit the secretion of proinflammatory neuropeptides from sensory afferents, namely calcitonin gene-related peptide (CGRP) and substance P, centrally and peripherally [21,22]. Targeting of BoNT/A specifically to sensory neurons could be beneficial for chronic pain alleviation by selectively disrupting neuropathic signalling pathways.

Cholera toxin has been shown to target certain populations of sensory neurons, but in nature, it infects the intestinal mucosa, leading to intense diarrhoea and dehydration via ionic imbalance and osmosis. The 84 kDa cholera toxin is produced by *Vibrio cholerae* as a complex of A and B subunits [23,24]. The cholera toxin B subunit (CTB) is a homo-pentamer forming the targeting portion of the toxin, which binds to GM1 gangliosides and fucosylated glycans on intestinal epithelial cell membranes [25]. The CTB-GM1 complex facilitates internalisation into the early endosomes, followed by retrograde translocation of the cholera enzymatic A subunit into the endoplasmic reticulum of the cell. The A subunit is made up of two chains, A1 and A2, which are attached by a disulfide bond. The A1 chain is the enzymatic portion of the toxin, and the A2 chain is a linker region bridging the enzymatic portion and the B subunit. When the toxin reaches the endoplasmic reticulum, the A1 and A2 chains are separated and the enzymatic A1 portion is translocated into the cytosol [26]. Recombinant CTB alone is non-toxic and can bind to a multitude of mucosal cells but also to peripheral neurons upon injection [27]. When labelled with fluorophores or other conjugates, CTB can serve as a neuronal tracer by its binding to GM1 gangliosides on neuronal membranes [28]. It was shown that only a subpopulation of sensory neurons is bound by CTB, and these are predominantly large-diameter, myelinated NF200-immunoreactive neurons [29,30,31].

We used a previously established SNARE-mediated linking strategy [32] to combine the cholera toxin binding domain with the botulinum type A enzyme. The linking technology was previously used to retarget the LcTd portion of BoNT/A to various neuropeptide receptors and to tetanus toxin receptors [33]. Here, the addition of the GM1-binding CT domain to the botulinum LcTd domain allowed targeting of a subpopulation of sensory neurons and also attenuation of mechanical allodynia in rat pain models with minimal effects on muscle function.

## 2. Results

### 2.1. Synthesis of ChoBot (Cholera-Botulinum Construct) and Its Functional Evaluation

**Figure 1:**
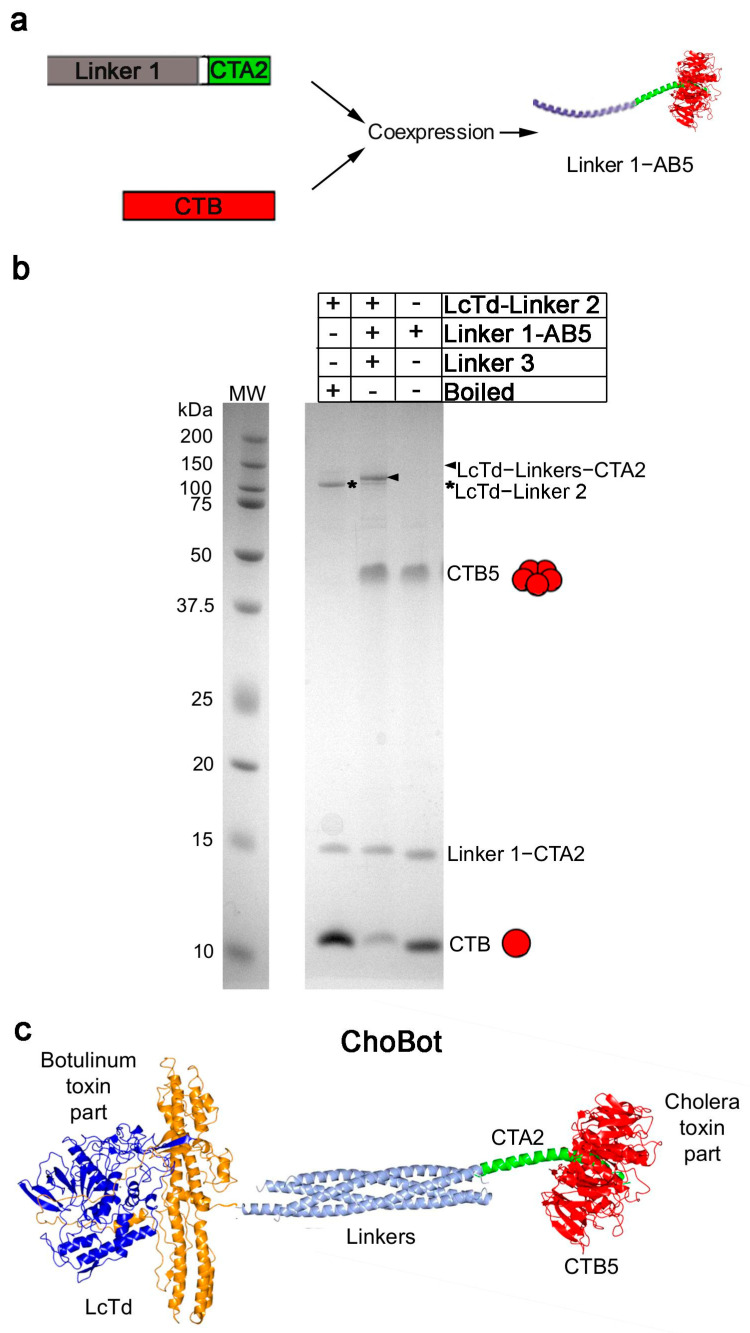
Production of ChoBot: (**a**) Schematic showing self-assembly of the AB5-linker, a CTB pentamer assembled around CTA2 fused to Linker 1, CTA2-cholera toxin A2 subunit, CTB-cholera toxin B subunits; (**b**) coomassie-stained SDS-PAGE gel showing that mixing the linker 1-AB5 and the light chain/translocation domain (LcTd) of BoNT/A attached to Linker 2 (LcTd-Linker 2) in the presence of Linker 3 (not visible due to low MW) results in formation of LcTd-Linkers-CTA2 (arrowhead). CTB5 is denoted as a pentamer of approx. 50 kDa. Note, the CTB5 and CTA2 association is not SDS-resistant and therefore only the linking of LcTd to CTA2 is visible as a slight shift in MW of LcTd-Linker 2. Boiling for 2 min in the SDS-PAGE sample buffer results in dissociation of CTB5 pentamer into monomeric B subunits. Molecular weight standards are denoted as MW; (**c**) schematic of ChoBot modelled using known X-ray structures of the BoNT/A, the SDS-resistant SNARE linking complex and cholera toxin (PDBs 3BTA, 1SFC and 1XTC, respectively).

The cholera toxin-related component, AB5 with linker 1, was produced by coexpressing two parts: (1) the cholera toxin A2 subunit (CTA2) fused to synaptobrevin and (2) the cholera toxin B subunits (CTB). During coexpression in *E. coli*, the CTB subunits form a pentamer around CTA2 (Figure 1a and Supplementary Figures S1 and S2) [34,35]. The formation of B5 pentamer was evident in the unboiled samples of the CTB protein, while the association of the CTB5 with CTA2-Linker 1 was evident by a faster migration of the protein complex in size-exclusion chromatography (Figure S2). The second required component was the botulinum toxin A protease with its translocation domain. This unit consists of the Light chain and Translocation domain (LcTd) attached to the SNAP25 linker 2 and was previously used for production of several functional botulinum constructs [32,33]. The third component, the syntaxin linker 3, functions as a ‘staple’. When all three components are mixed for 60 min, the three linkers self-assemble into an SDS-resistant coiled coil that can be observed by a shift in migration of the 125 kDa LcTd-Linker 2. Since in SDS the B5 pentamer does not retain its association with CTA2 [34,35], only a slight shift in the migration of the 125 kDa LcTd-Linker 2 was evident (Figure 1b, the arrowhead). This shift confirms that the ‘stapling’ step was successful. Given that the underlying AB_5_ complex is known to assemble correctly (Figure S2c) and remains stable in non-denaturing conditions [34], we conclude that ChoBot is successfully formed. The nearly complete shift observed in the migration of LcTd-Linker 2 in the SDS-PAGE gel in the presence of the 10-fold molar excess of the AB5-linker 1 allowed calculation of the amount of the formed toxin chimaera in the reaction mix (named ChoBot) (Figure 1b,c).

**Figure 2:**
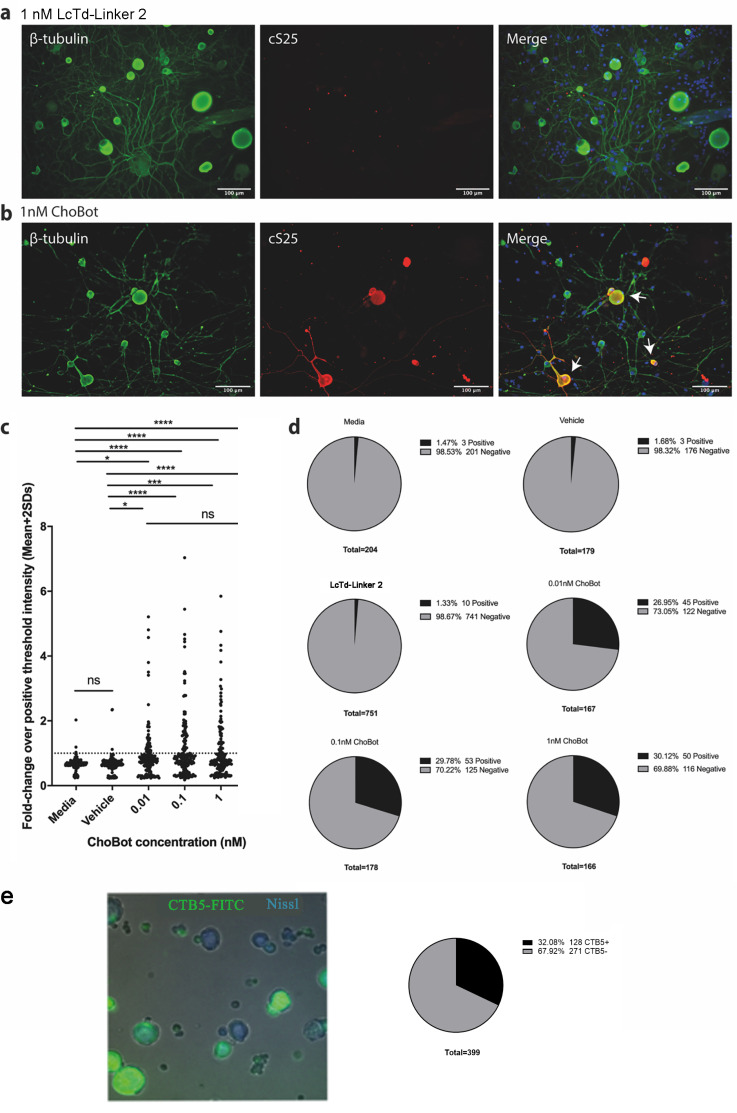
ChoBot cleaves SNAP25 in a subpopulation of primary cultured rat dorsal root ganglion (DRG) neurons. Representative immunocytochemistry images of cleaved SNAP25 (cS25) in DRG neurons co-labelled with the pan-neuronal marker beta-tubulin in DRG cultures treated with 1 nM LcTd-Linker 2 (**a**) or 1 nM ChoBot (**b**). The arrows indicate colocalised labelling. (**c**) Cleaved SNAP25 intensity measurements from betaIII-tubulin positive neurons following treatment without or with different concentrations of Chobot. Cells were deemed positive for cleaved SNAP25 with intensities over the mean + 2 standard deviations of intensity measurements of control wells from the same experiment. In total, 6 images per well, 2 wells per condition, and 2 separate cultures. One-way ANOVA followed by Tukey’s multiple comparisons test. * = *p* ≤ 0.05, *** = *p* ≤ 0.001, **** = *p* ≤ 0.0001, ns: Not Significant. (**d**) Pie charts showing the proportion of DRG neurons classed as positive for cleaved SNAP25. (**e**) Representative image of rat DRG neurones stained with fluorescent CTB5-FITC product (Merck, Gillingham, UK, cat. No. C1655) (left) with the pie chart showing the proportion of CTB5-labelled neurons (right). Neuronal-specific marker Nissl was used to determine the total neuronal count. Of a total of 399 DRG neurons, 32 ± 6.2% (mean ± SD) were positive for CTB5.

To evaluate the functionality of ChoBot, we investigated its targeting of rat dorsal root ganglion (DRG) neurons. We applied ChoBot at 0.01–1 nM concentration to cultured DRG neurons for 24 h and estimated the percentage of cells exhibiting botulinum-cleaved SNAP25 as revealed by immunocytochemistry utilising an antibody specifically recognising the botulinum-cleaved end of SNAP25. Figure 2a,b shows that ChoBot triggers cleavage of SNAP25 in cultured DRG neurons (27–30% of total estimated using the pan-neuronal βIII-tubulin). In contrast, LcTd-Linker 2 on its own exhibited minimal cleavage of neuronal SNAP25 (<2%). DRG cultures were also labelled with a commercially available fluorescent CTB5 product widely used to visualise and trace GM1-containing cells. Evaluation of the number of fluorescently labelled cells as a percentage of total cell count, as measured by the Nissl neuronal staining, demonstrated that 32% of rat DRG neurons in culture are targeted by CTB5 (Figure 2e). These results together show that the addition of AB5 to LcTd allows a nearly complete coverage of GM1-containing sensory neurons, followed by functional cleavage of SNAP25 in rat DRG cultures.

### 2.2. Testing of ChoBot in Rat Pain Models

**Figure 3:**
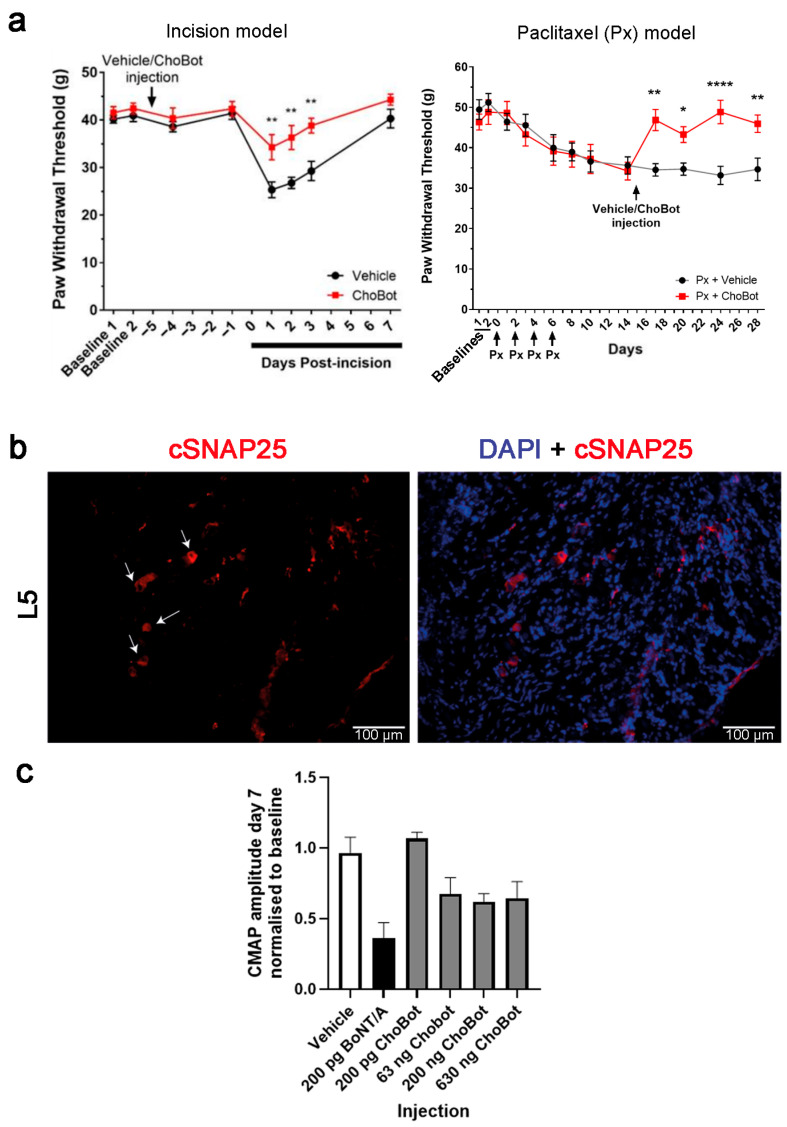
ChoBot prevents and reverses mechanical allodynia, respectively, in a surgical incision and a neuropathic model: (**a**) Graphs showing electronic von Frey mechanical threshold measurements following either a single preemptive ChoBot injection in a footpad incision model (left) or a single ChoBot footpad injection in a paclitaxel-induced neuropathic model (right). Vehicle control injection (black), ChoBot injection, 200 ng (red). Two-way ANOVA, +/− SEM. * = *p* ≤ 0.05, ** = *p* ≤ 0.01, **** = *p* ≤ 0.0001 (**b**) Representative immunohistochemical images of cleaved SNAP25 (red) in the L5 DRGs of 200 ng ChoBot-injected naïve rats. Arrows denote cleaved SNAP25-immunoreactive soma. (**c**) Bar chart showing CMAP amplitudes of supramaximally stimulated rat gastrocnemius muscle 7 days following a subcutaneous injection of Chobot at indicated doses or BoNT/A at 200 pg over the recording site. The amplitude of the CMAP post-injection was normalised to each individual animal’s baseline CMAP amplitude pre-injection. N = 3, one-way ANOVA followed by Tukey’s multiple comparisons test. Data presented as mean ± SEM.

Behavioural testing following ChoBot injection into the plantar surface of the hind paw was carried out using electronic von Frey to measure the paw withdrawal threshold in postoperative and chemotherapy rat pain models. The first pain model was a plantar incision, a model of post-operative pain with both neuropathic and inflammatory components. An injection of 200 ng ChoBot 5 days prior to the incision significantly reduced the transient mechanical allodynia that developed as a result of the incision (Figure 3a). The second model was a chemotherapy-induced peripheral neuropathy model induced by 4 injections of paclitaxel over 8 days. A subsequent injection of ChoBot into the hind paw reversed developed mechanical allodynia on the ipsilateral side (Figure 3b). Therefore, ChoBot is functionally active in silencing nociceptive pathways and shows preclinical efficacy in the treatment of pain with neuropathic components. We analysed whether cleaved SNAP25 can be detected in the DRGs extracted 5 days after plantar injection of ChoBot. Figure 3c and Supplementary Figure S3 show that cleaved SNAP25 immunoreactive cell bodies were detectable in the lumbar L3–L5 DRGs innervating the sciatic nerve, with L5 showing the highest level of SNAP25 cleavage. Intraplantar injection of 200 ng ChoBot did not produce overt muscle paralysis as observed over 4 weeks. To confirm this lack of potent paralysis, we performed CMAP measurements directly comparing ChoBot and BoNT/A. Rats were injected subcutaneously with 30 μL of ChoBot at different doses or BoNT/A at 200 pg over the recording site of the left gastrocnemius muscle. Figure 3c shows that ChoBot at a 1000 higher dose did not affect CMAP to the same degree as BoNT/A, confirming it lacks the paralytic potency of the native toxin.

**Figure 4:**
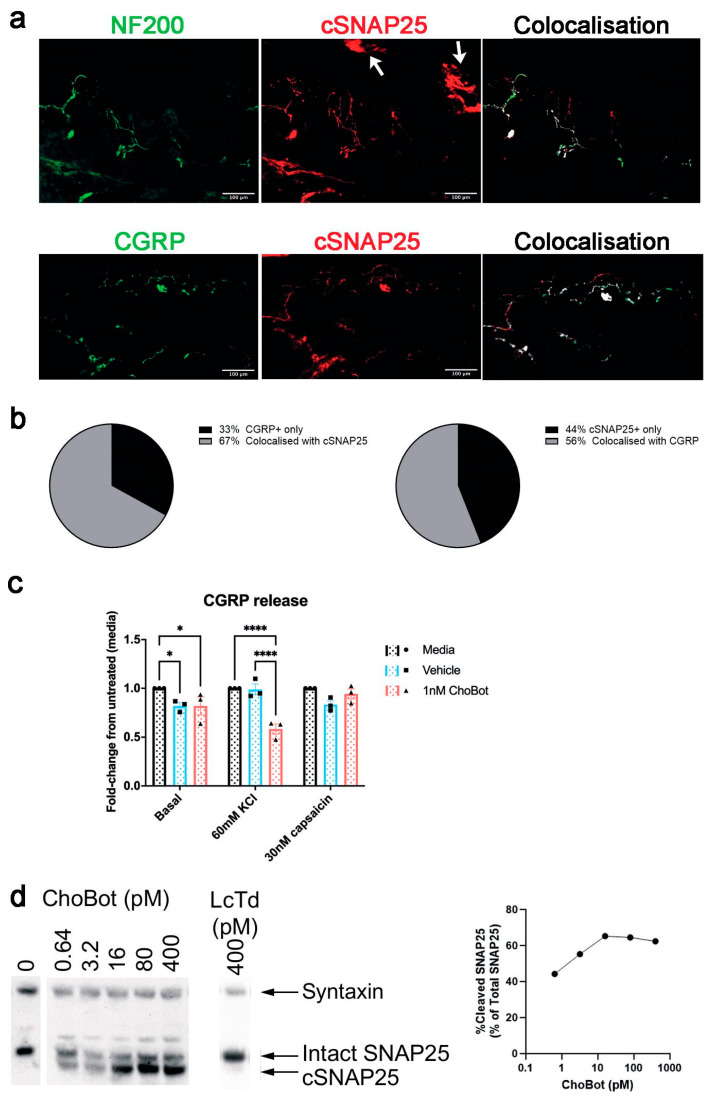
ChoBot cleaves SNAP25 in the skin tissue and inhibits release of CGRP in DRG cultures. (**a**) Representative immunohistochemical images of cleaved SNAP25 in rat skin neuronal fibres. Skin was taken from the hind footpad 1 week after intraplantar injection of 200 ng ChoBot. Colocalisation with NF200- and CGRP-positive neuronal fibres is highlighted on the right side. The arrows indicate a non-specific staining of stratum corneum. N = 2, 5 images analysed per N; (**b**) quantification of colocalisation of CGRP and ChoBot-targeted skin neuronal populations; (**c**) bar chart showing ChoBot-induced inhibition of KCl-triggered but not capsaicin-triggered CGRP release in rat DRG neuronal cultures. Two-way ANOVA of treatment and stimulus followed by Tukey’s multiple comparisons test. N = 3, data presented as mean ± SEM. * = *p* ≤ 0.05, **** = *p* ≤ 0.0001 (**d**) Immunoblot (left) and band quantification data (right) showing that ChoBot but not LcTd triggers cleavage of SNAP25 in the picomolar range in rat DRG neurons. Note that a proportion of SNAP25 remains intact at higher ChoBot concentrations. Syntaxin immunostaining was used as a loading control.

Next, immunohistochemistry of skin sensory fibres was performed following subcutaneous injection of ChoBot into the plantar surface of the hind paw. Immunoreactivity for cleaved SNAP25 was detectable in dermal and epidermal fibres at the site of injection and these colocalised with markers of sensory neurons, with significant colocalisation of cleaved SNAP25 in NF200-expressing fibres and CGRP-expressing fibres (Figure 4a). Quantification of colabelling revealed that 67% of CGRP-positive skin fibres exhibit ChoBot-cleaved SNAP25, with 56% of ChoBot-sensitive skin fibres exhibiting CGRP co-staining (Figure 4b). The robust labelling of CGRP fibres led us to investigate whether the application of ChoBot to DRG cultures can inhibit CGRP release. DRG cultures were pretreated with ChoBot or vehicle for 18 h and then the release of CGRP was triggered using depolarisation with 60 mM KCl or TRPV1 activation with 30 nM capsaicin. An ELISA assay of the DRG culture supernatants revealed that ChoBot inhibited only depolarisation-triggered CGRP release but not capsaicin-induced CGRP release. This further suggests a focused action of ChoBot on particular sensory neuronal subpopulations (Figure 4c). Western immunoblotting of DRG cultures demonstrated that ChoBot, even at low picomolar doses, can efficiently cleave SNAP25. A proportion of SNAP25 remained intact even at 400 pM, indicating that some populations of DRG neurons are less sensitive to the action of ChoBot (Figure 4d).

**Figure 5:**
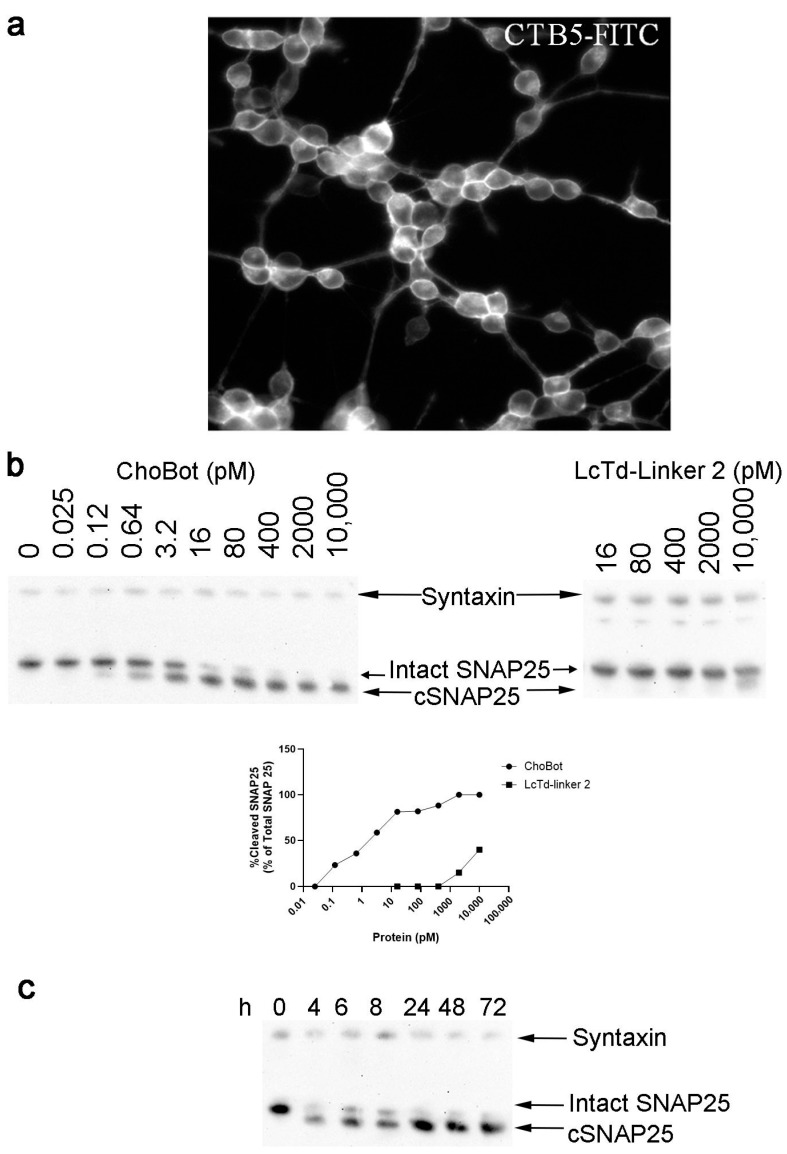
Functional evaluation of ChoBot in human neuroblastoma cell cultures: (**a**) representative image of neuronally differentiated SiMa cells stained with fluorescent CTB5-FITC; (**b**) immunoblots (top) and band quantification (bottom) showing ChoBot-triggered cleavage of SNAP25 in the picomolar range in differentiated SiMa cells, which is not observed for LcTd-linker 2. Note almost complete cleavage of SNAP25 at 16 pM of ChoBot, whereas no SNAP25 cleavage has occurred when LcTd-linker 2 is added without AB5. (**c**) Immunoblot showing the time course of the cleavage of SNAP25 in differentiated SiMa cells treated with 1 nM ChoBot. Syntaxin immunostaining was used as a loading control.

To evaluate the functionality of ChoBot in human cells, we investigated SiMa neuroblastoma neurons, which were introduced as an efficacy cell model for testing commercial BoNT/A preparations [36]. Application of fluorescent CTB5-FITC to differentiated SiMa cells resulted in a robust labelling of all SiMa cells, suggesting GM1 ganglioside presence in this neuronal model (Figure 5a). Application of ChoBot to the cell cultures for 48 h led to the complete cleavage of SNAP25 in the picomolar range as revealed by immunoblotting using total SNAP25 antibody, with an EC50 of ~3 pM (Figure 5b). Investigation of the kinetics of SNAP25 cleavage revealed that cleaved SNAP25 appears 4 h after application of ChoBot (Figure 5c).

## 3. Discussion

Neuropathic pain is defined as pain that arises from damage or disease of the somatosensory nervous system [37]. It has many diverse causes and can arise from lesions or disease of either the central or peripheral somatosensory system. Traumatic damage can induce neuropathic pain peripherally, but the most common cause of peripheral neuropathy is through disease. One common peripheral neuropathy is painful diabetic neuropathy, with approximately half of diabetic patients experiencing it in some form [38]. Viral infections can also lead to chronic neuropathic pain, as in the case of postherpetic neuralgia following resolution of the herpes zoster virus [39]. Chemotherapy drugs are also toxic to the peripheral sensory neurons and neuropathic pain can develop during or following cancer treatment. The incidence of chemotherapy-induced peripheral neuropathy (CIPN) is estimated to be as high as 60% three months after the cessation of chemotherapy [40]. Given the high prevalence of neuropathic pain and its substantial impact on quality of life, there is a clear need for effective treatments [3].

Peripheral treatments for peripheral neuropathies may offer advantages by avoiding the adverse effects associated with centrally acting analgesics. Botulinum neurotoxin type A injections have demonstrated benefit in treating chronic migraine and have also shown some efficacy in neuropathic pain [17,41,42]. Suggested mechanisms of BoNT-mediated analgesia include direct silencing of neurotransmission from the DRG neuron to the spinal cord, prevention of the release of pro-inflammatory peptides such as substance P and CGRP in the periphery, and the prevention of pain-mediating ion channels being trafficked to the membrane [21,22,43,44,45]. BoNT injections have a therapeutic advantage over other pain treatments, as a single injection session has effects that last for months due to BoNT’s long-lasting effect on SNARE proteins [46]. Unfortunately, BoNT/A provides suboptimal analgesia, as response rates and degrees of pain relief remain limited, in part due to the dose constraints imposed by paralytic side effects. Our previous attempts to reduce muscle paralysis, while preserving the sensory activity of BoNT/A, relied on structural modifications [33]. The elongated BoNT/A variants demonstrated substantially lower muscle paralysis while being efficient in alleviating mechanical allodynia in several pain models [47,48,49]. However, these new constructs still utilised the limited affinity of BoNT/A to sensory neurons. Other retargeting strategies involved replacement of the BoNT/A receptor-binding domain with neuropeptide substance P or a morphine analogue, but these required central injections of the retargeted toxin [50], limiting the utility of neuropeptide-toxin constructs in the clinic.

Here, we demonstrate that ChoBot, a construct of cholera toxin and botulinum neurotoxin, has the targeting properties of cholera and the SNAP25-cleaving properties of BoNT/A. Our findings show that ChoBot effectively cleaves SNAP25 in sensory neurons both in vitro and in vivo. In cultured DRG neurons, ChoBot affected a subpopulation of neurons, with cleaved SNAP25 being detected in approximately a third of the neurons. As culture conditions may not accurately reflect the physiology of sensory neurons in vitro, injections of ChoBot into the plantar surface of the hind paw, with subsequent analysis of targeted neuronal populations, were also performed. After ChoBot injection, cleaved SNAP25 was observed in sensory neurons of the dermis and epidermis as well as in the cell bodies of the DRG, demonstrating that ChoBot is functional in vivo and that its action is not limited to its site of injection. In paw tissue, SNAP25 cleavage was most prominent in NF200-positive fibres, but also in a substantial proportion of CGRP-expressing fibres. Within the lumbar DRG, SNAP25 cleavage was observed mostly in the L5 segment, consistent with innervation of the injection site by the sciatic nerve [51]. The pattern of susceptibility, with strong cleavage in NF200-positive large-diameter neurons and detectable cleavage in CGRP-positive populations in vivo, is consistent with known GM1 distribution and supports the conclusion that ChoBot enters neurons via GM1-mediated binding and internalisation [29,30,31]. ChoBot contains the cholera toxin B subunit together with a C-terminal KDEL motif on the A2 peptide, features known to drive retrograde trafficking from the Golgi to the endoplasmic reticulum (ER) [26]. In addition, ChoBot incorporates the BoNT/A translocation domain, which mediates escape of the metalloprotease from acidified early endosomes [10]. While endosomal acidification is widely accepted as the primary mechanism enabling BoNT/A light chain translocation into the cytosol, recent work has identified an alternative Sec61dependent pathway in which the metalloprotease is released from the ER into the cytosol [52]. Thus, ChoBot could theoretically use either, or both, routes for cytosolic entry. Quantifying the proportion of ChoBot that follows classical BoNT-like early endosomal escape versus ER-based retrograde trafficking is beyond the scope of the current study. Nonetheless, the observed cleavage of SNAP25 both in vitro and in vivo demonstrates that internalization of ChoBot enables the obligatory escape of the botulinum enzyme into the neuronal cytosol.

Behavioural testing demonstrated a functional effect of ChoBot injection in vivo. Two different rat models of pain were used to induce mechanical allodynia, assessed by electronic von Frey as a reduction in the mechanical threshold for paw withdrawal. The first was a plantar incision, as a model of postoperative pain with both inflammatory and neuropathic elements [53]. As the reversal of mechanical allodynia following this incision is over the course of only a few days, a preemptive injection of ChoBot was given five days prior to the incision to allow maximal SNARE cleavage to occur. ChoBot significantly reduced mechanical hypersensitivity over the days following incision. The second model used was a model of CIPN using the anti-cancer drug paclitaxel. Over the course of four injections of 2 mg/kg paclitaxel, mechanical allodynia developed in both hind paws. A subsequent injection of ChoBot into one of the paws demonstrated a significant reversal of mechanical allodynia ipsilaterally. Our study demonstrates that the chimeric ChoBot can reverse pain behaviours in two animal models of nerve injury by targeting a large subpopulation of DRG neurons overlapping with pain-related neuropeptidergic nociceptors. Future studies will need to address the dose- and time-dependent effects of highly purified ChoBot in pain models. To this end, it will be important to employ covalent linkage between the botulinum and cholera toxin components, as enabled by SpyCatcher technology, for the production of the ChoBot chimaera [49].

Interestingly, subcutaneous injections of 200 ng ChoBot did not elicit obvious muscle paralysis. Indeed, further CMAP measurements indicated that ChoBot, even at very high doses, cannot reach the same inhibition of muscle function as observed with BoNT/A at picogram quantities. The observed lack of muscle paralysis after ChoBot injection is intriguing, considering that motor neurons can bind cholera toxin binding domain albeit with typical intramuscular injection doses used at 10 micrograms, and with immunological tracing requiring even higher doses [54]. As GM1 is widely expressed on many non-neuronal cell types, a portion of the injected ChoBot is likely sequestered locally by these GM1-positive cells, reducing the amount that ultimately reaches motor neurons. Any such off-target uptake, together with the nanogram doses used and the subcutaneous route of injections typical for analgesic studies of BoNT/A [33,55], likely contributes to the absence of observable muscle paralysis. While any off-target uptake could potentially be associated with other adverse off-target effects, none were observed in our study.

In summary, this study reports that a cholera toxin-binding domain, when combined with the light chain/translocation domain of BoNT/A, can direct the botulinum proteolytic activity to rat sensory neurons and human neuroblastoma cells. The resulting toxin combination robustly cleaves SNAP25 in a subpopulation of sensory neurons and reverses mechanical allodynia in neuropathic pain models without the strong paralytic effects of native BoNT/A, thereby unveiling a new strategy for long-term neuronal silencing in chronic pain.

## 4. Materials and Methods

### 4.1. Animals

All procedures were conducted under UK Home Office Project, Personal, and Institutional Licenses and complied with the UK Animals (Scientific Procedures) Act, 1986. The guidelines for pain research in animals published by the International Association for the Study of Pain [56] were followed. Animal numbers were determined by power calculations using data from previous experiments employing similar protocols. Experiments were conducted on male Sprague-Dawley rats aged 6–10 weeks purchased from Charles River (UK). All animals were housed in a 12/12 h light/dark cycle at 21 °C and in 55% relative humidity. Food and water were available ad libitum. A total of N = 5–6 rats per group were used for behavioural experiments, N = 3 for electromyography, and N = 2 for immunohistochemistry.

### 4.2. Preparation of Botulinum Neurotoxin–Cholera Toxin Conjugates

Preparation of ChoBot consisted of production and purification of three independent recombinant units that were subsequently assembled by SNARE-mediated stapling: (1) the BoNT/A light-chain–translocation domain fused to SNAP25 (LcTd/A–Linker 2); (2) a synthetic syntaxin helix peptide (AEDAEIIKLENSIRELHDMFMDMAMLVESQGEMIDRIEYNVEHAVDYVE); and (3) the cholera toxin binding domain fused to the Synaptobrevin helix (Linker 1-AB5). The synthetic syntaxin peptide was commercially synthesised (Peptide Synthetics, Fareham, UK). The LcTd/A-Linker 2 was expressed in the BL21 strain of *E. coli* as a glutathione S-transferase N-terminal fusion. Protein fused to glutathione S-transferase was purified on Glutathione Sepharose beads (GE Healthcare, Amersham, UK) and eluted in 20 mM HEPES, pH 7.3, 100 mM NaCl (Buffer A) using thrombin (1 U/L of culture). Further purification was achieved by gel filtration using a Superdex 200 10/200 GL column (GE Healthcare, Amersham, UK).

The Linker 1-AB 5 binding domain was expressed from a bicistronic periplasmic expression plasmid based on pSAB2.1 [34] modified to have a codon-optimised sequence for Linker 1-synaptobrevin (residues 2–84, Rattus norvegicus) and GGNNS spacer between the TEV protease cleavage site and the C-terminal 25 residues of cholera toxin A2 (CTA2) (Supplementary Figure S1). The resulting plasmid (pSAB-Linker 1-AB 5) allowed co-expression of MBP-Linker 1-CTA2 with GGG- which assembles into the MBP-Linker 1-AB5 binding domain (Supplementary Figure S2). The MBP fusion was purified by successive amylose and Ni-NTA affinity chromatography to select only proteins with both A- and B-subunits (Supplementary Figure S2). The MBP tag was then removed by TEV protease digestion, and the reaction mixture was subjected to a second Ni-NTA step to separate cleaved MBP and MBP-TEV protease from the Syb-AB5 complex. While CTB typically appears as a ~50 kDa pentamer on SDS-PAGE if the sample has not been boiled, the CTA2 association with CTB5 is not stable under standard SDS-PAGE conditions [34,35]. SDS-PAGE analysis of boiled and unboiled samples of MBP-Syb-AB5 and Syb-AB5 demonstrated successful formation of the AB5 complexes while size-exclusion chromatography provided evidence for the formation of Syb-AB5 (Supplementary Figure S2b,c).

The full ChoBot construct was assembled by mixing the three components for 60 min with a 10-fold excess of AB5 over the LcTd-Linker 2 at 20 °C in Buffer A containing 0.4% beta-octylglycoside, OG. After confirmation of the assembly by SDS-PAGE and Coomassie staining (Figure 1b), the protein was aliquoted and stored at −80 °C before functional experiments. The concentration of LcTd-linker 2 in the reaction was known to be 200 nM, so as all of the LcTd-linker 2 was combined with linker 1-CTA2 after the reaction, the presumed molar concentration of LcTd-linkers-CTA2 (and ChoBot in solution) would also be 200 nM.

Native BoNT/A was from Metabiologics, Lot number A042519-01.

### 4.3. Primary Culture of Dorsal Root Ganglion Neurons

For DRG cultures, rats (3–8 weeks) were anaesthetised in isofluorane and sacrificed by cervical dislocation, with confirmation by decapitation and exsanguination. Dorsal root ganglia (DRG) from all vertebral levels were harvested into PBS. The DRGs were digested for 1 h in a mixture of 0.6 mg/mL collagenase type IX (Merck, Gillingham, UK) and 1 mg/mL Dispase II (Gibco, Paisley, UK) in PBS at 37 °C in 5% CO_2_ and 95% air. Following trituration, the cell suspension was layered onto 15% BSA in DMEM/F12 and centrifuged at 800× *g*. The pellet was resuspended in the following serum-free, neuronal-specific medium before plating: Neurobasal A (ThermoFisher, Loughborough, UK), penicillin (100 units/mL) and streptomycin (100 µg/mL) (Merck, Gillingham, UK), NGF2.5S (20 ng/mL Merck, Gillingham, UK), Glutamax (1%, Gibco, Paisley, UK), B27 (1×, ThermoFisher, Loughborough, UK), Uridine and 5-Fluoro-2′-deoxyuridine (both 20 µM, Merck, Gillingham, UK). For labelling and ELISA experiments, DRG neurons were plated in 96-well µClear plates (Greiner, Stonehouse, UK) coated with laminin (Merck, Gillingham, UK). For Western blot, neurons were plated onto laminin-coated 48-well plates. For CTB5 binding studies, experiments were performed on day (0) of the culture to minimise proliferation of non-neuronal cells, but at least 3 h after seeding to allow cells to adhere to the bottom of the dish. For experiments with ChoBot, DRG cells were grown for three days prior to toxin treatment. Non-treated control wells were included for all experiments.

### 4.4. SiMa Cell Culture and Differentiation

SiMa cells (DSMZ) were sub-cultured in growth media of RPMI media (Life Technologies, Paisley, UK) supplemented with 10% Foetal Bovine Serum (FBS) (Life Technologies, Paisley, UK) and passaged with Trypsin-EDTA according to the supplier protocols. Cells were maintained at 37 °C, 5% CO_2_ and not used for more than 20 passages (10 weeks). For differentiation, trypsinised cells in growth medium were centrifuged at 3000 RPM for 5 min and resuspended in differentiation medium: Neurobasal medium (ThermoFisher, Loughborough, UK), 1X B-27 (Invitrogen, Paisley, UK), 1 mM HEPES, pH 7.2 (ThermoFisher, Loughborough, UK) with 10 µM All-trans retinoic acid (Merck, Gillingham, UK). For labelling with CTB-488, cells were plated in 96-well µClear plates (Greiner, Stonehouse, UK) coated in laminin (Merck, Gillingham, UK) at a density of 1 × 10^4^ cells per well. For Western blotting, cells were seeded at a density of 1 × 10^5^ cells per well in 48-well plates. In all cases, wells were coated with laminin (Merck, Gillingham, UK) and cells were differentiated for 72 h before functional studies.

### 4.5. Immunocytochemistry and Quantification

Wells were washed once with ice-cold PBS for 2 min, then fixed for 10 min with ice cold 4% PFA in PBS. The cells were then permeabilised with 0.1% triton X-100 for 15 min. Blocking solution consisting of 2% fish skin gelatin, 2% BSA and 0.1% Tween-20 in PBS was added to the wells at room temperature for 1 h. Primary antibodies were diluted in blocking solution and then applied to the wells for 1 h at room temperature. Primary antibodies were washed three times with PBS. Fluorescent secondary antibodies, CTB-FITC (Merck, Gillingham, UK) and DAPI were diluted in blocking solution and applied to the cells for 45 min at 20 °C in the dark before imaging. The cells were incubated with Neurotrace Blue Fluorescent Nissl stain (1:500, ThermoFisher, Loughborough, UK), where indicated, before three washes prior to imaging. Imaging was performed on an InCell Analyser 2200 (GE Healthcare, Amersham, UK) or manually using a Leica DMi8 microscope, Leica DFC3000G camera and CoolLED pE-300 Ultra light source using Leica Application Suite X (Leica, Milton Keynes, UK). Image analysis and quantification were performed using FIJI (v1.5). Circular regions of interest (ROIs) were drawn around cell bodies of neurons (as marked by either pan-neuronal markers or markers of certain neuronal populations). The Multi-Measure function was then used to measure the fluorescent intensities (either mean grey value or min and max grey value, depending on the experiment) of the relevant wavelength of the image. These were then copied into Microsoft Excel for analysis. Thresholds for positivity were set by measuring the fluorescent intensities of cell bodies of a negative control for each culture (i.e., wells that were not treated with toxin for cleaved SNAP25 quantification). A threshold was then set for each culture at the mean + 2 standard deviations of the negative control intensities measured.

### 4.6. CGRP Release Assay

CGRP release was assayed using an acetylcholinesterase-based enzyme-linked immunoassay (ELISA) kit from Bertin Bioreagent (Montigny le Bretonneux, France) (A05482). Briefly, the wash buffer, enzyme immunoassay buffer and anti-CGRP tracer were made according to the kit instructions. Stimulating compounds were made up at working concentrations in HEPES-buffered Tyrodes solution (HBTS) with 10 μM DL-Thiorphan (Bachem, Bubendorf, Switzerland, N-11950025) and warmed to 37 °C. A CGRP standard concentration curve was made up in enzyme immunoassay buffer from the highest concentration of 500 pg/mL with seven subsequent 1:2 serial dilutions. Just before stimulation, ELISA plates were washed five times with wash buffer, then 100 μL of anti-CGRP tracer was added to each well. The CGRP concentration curve was added to the relevant wells. Media was removed from DRG neurons in a 96-well plate. Wells were washed once with warmed HBTS and thiorphan, then warmed compounds were added to the wells, and the plate was incubated at 37 °C for 10 min. The supernatant was then carefully removed from the wells and placed into the ELISA plate with the tracer. ELISA plates were sealed and placed at 4 °C overnight. On the second day, Ellman’s reagent was made up per the kit instructions. ELISA plates were washed five times with wash buffer, and then 200 μL of Ellman’s reagent was added. The plates were left to develop at room temperature on an orbital plate shaker in the dark for between 30 and 60 min. Absorbance was then measured at 405 nm using a plate reader (FLUOstar Optima, BMG LabTech, Ortenberg, Germany). The mean absorbance of the blank well controls was subtracted from the absorbance of each well and the optical density of the standard curve was plotted to convert the absorbance into CGRP concentration.

### 4.7. Compound Muscle Action Potential Electromyography

Rats were anaesthetised with 4% isoflurane and maintained at 2%. Stimulating needle electrodes (ELSTM2; Biopac, Goleta, CA, USA) were inserted perpendicularly into the muscle approximately 0.5 cm from the fifth lumbar vertebrae on either side. The anode was always placed distally and the cathode placed proximally to the recording leg. A ground electrode (EL452, Biopac, Goleta, CA, USA) was placed at the base of the tail. A reference recording needle electrode (EL450, Biopac, Goleta, CA, USA) was placed over the tendon of the gastrocnemius muscle, and a recording electrode was placed in the belly of the medial gastrocnemius muscle. CMAP measurements were performed using a Biopac system with a bandpass of 30–9999 Hz and 200× gain, running AcqKnowledge software (100W). A 0.2 ms pulse stimulation was performed with a voltage stimulator (BSLSTMB). Supramaximal stimulation was determined for each recording. The amplitude of the CMAP waveform was then measured. Eight recordings per leg were performed, replacing the recording electrode each time, and the largest three recordings were averaged. Baseline CMAP recordings were determined for the gastrocnemius of each hind limb. Rats were then each injected with 30 μL of a botulinum construct subcutaneously over the recording site of the left gastrocnemius muscle using a BD Micro-Fine 0.5 mL insulin syringe immediately after baseline recording. All injections and recordings were performed blinded to the toxin and concentration given. CMAPs were then recorded from both gastrocnemius muscles again on days 1, 2, 3, and 7, and the fold-change to the baseline CMAP at each time point was calculated for each rat. At the end of the experiment, the animals were sacrificed by the Schedule 1 method.

### 4.8. Pain Models and Von Frey Testing

Two experimental pain models were employed. The first was the plantar incision model, representing postoperative pain with both inflammatory and neuropathic components, as originally described by Brennan et al. [53]. The second was a paclitaxel-induced CIPN model [57]. Paclitaxel was dissolved to 6 mg/mL in a 1:1 ratio of Kolliphor EL (C5135, Merck, Gillingham, UK) and ethanol (E/0665DF/17, Fisher Scientific, Loughborough, UK). Aliquots were then frozen for storage at −20 °C if required. The paclitaxel was then diluted to 2 mg/mL in 0.9% saline solution (12446-01, Dechra, Shrewsbury, UK). Rats were anaesthetised with 4% isoflurane in oxygen in an induction chamber for paclitaxel injections. Injections were given at 2 mg/kg via an intraperitoneal injection using a 23-gauge needle coupled to a 1 mL syringe. Four injections were given every other day over eight days, to give a total cumulative dose of 8 mg/kg per rat. Vehicle groups received equivalent injections of Kolliphor EL/ethanol/saline. Animals were observed until they recovered from anaesthesia.

Behavioural testing was conducted by an experimenter blinded to the treatment group. Static mechanical hypersensitivity was assessed using an electronic von Frey test (Harvard Apparatus, Holliston, MA, USA) according to a previously established protocol [58]. Rats were placed in individual chambers positioned above a wire mesh grid and allowed to acclimatise for 30 min prior to testing. The force transducer tip was gently applied to the central plantar surface of the hind paw from below, and pressure was increased gradually until a brisk paw withdrawal response was observed. The paw withdrawal threshold (in grams) was automatically recorded by the device. Testing was performed between 9:00 and 12:00 each day, when animals were calm but awake. A minimum of three consecutive measurements were taken for each paw, and the mean value was used for analysis.

### 4.9. Immunohistochemistry and Imaging

Rats were sacrificed by cervical dislocation, and lumbar-dorsal root ganglia and hind paw skin were dissected [59]. DRG were fixed in 4% PFA in PBS at 4 °C for 2–4 h before being placed at 4 °C overnight in 30% sucrose in PBS. The hind paw skin was fixed in Zamboni’s solution (a phosphate-buffered solution of 2% PFA and 15% picric acid) before being cryoprotected in the same way. For sectioning, tissue was embedded in Cryo-M-Bed OCT medium (53581-1, Bright) and frozen on dry ice. Sections were prepared using a cryostat (OFT5000, Bright Instruments, Huntingdon, UK). DRG were sectioned at 13 μm thickness and collected directly onto SuperFrost Plus slides (ThermoFisher, Loughborough, UK) and stored at −20 °C until use. Skin sections were cut at 30 μm and collected as free-floating sections into cryoprotectant solution and stored at −20 °C until use.

Immunohistochemistry was all performed at room temperature. After PBS washes and 1 h blocking, sections were incubated with primary antibodies diluted in blocking buffer for 2 h, followed by Alexa Fluor-conjugated secondary antibodies and DAPI for 2 h in the dark. Slides were then coverslipped using Fluoromount-G mounting medium (00-4958-02, Invitrogen) and #1.5 thickness 24 mm x 60 mm coverslips (ThermoScientific, Loughborough, UK 631-0853). Tissue sections were imaged using a Leica 20× air objective (numerical aperture 0.4), Leica DMi8 microscope, Leica DFC3000G camera and CoolLED pE-300 Ultra light source (Leica, Milton Keynes, UK). Images were acquired using Leica Application Suite X (Leica, Milton Keynes, UK). Co-localisation analysis was performed in FIJI. The Colocalisation Threshold function was applied.

### 4.10. Western Immunoblotting

Various concentrations of either ChoBot or LcTd-linker2 were added to the plated DRG cultures (Methods 4.3) or differentiated SiMa (Methods 4.4) for 3 days at 37 °C in 5% CO_2_ and 95% air. Cell culture medium was then removed and cells were lysed in 45 μL of SDS-PAGE running buffer (56 mM sodium dodecyl sulphate, 0.05 M Tris-HCl, pH 6.8, 1.6 mM EDTA, 6.25% glycerol, 0.0001% bromophenol blue, 10 mM MgCl2, 26 U/mL benzonase) by shaking at 900 rpm for 10 min. Lysed cells were then boiled at 95 °C for 3 min and then run on 12% Novex SDS–PAGE gels (Invitrogen) for 3 h at 4 °C to increase separation between cleaved and intact SNAP-25. Following separation, proteins were transferred onto Immobilon-P membranes, and then incubated for 30 min in blocking solution (5% milk, 0.1% Tween 20 in PBS). The rabbit SNAP-25 polyclonal antibody (1:3000 dilution) and rabbit polyclonal Syntaxin antibody (1:1000 dilution) were added to the blocking solution at 4 °C overnight. Membranes were washed three times in 0.1% Tween 20 in PBS for 5 min and then incubated for 30 min in the blocking solution containing secondary peroxidase-conjugated donkey anti-rabbit antibodies (Amersham). Membranes were washed three times for 5 min in 0.1% Tween 20 in PBS. Immunoreactive protein bands were visualised using SuperSignal West Dura solution (Thermo Scientific, Loughborough, UK) on a BioRad imaging station and by exposure to X-Ray films (Hyperfilm MP, GE Healthcare, Amersham, UK). Protein bands were quantified using ImageJ 1.54g software. Quantification of band densities are presented in Supplementary Table S1.

### 4.11. Antibodies

βIII-tubulin (rabbit polyclonal, MAB1195, R&D systems (Abingdon, UK)); CGRP (mouse polyclonal, C7113, Merck, Gillingham, UK). Cleaved SNAP25 (rabbit polyclonal), SNAP25 (rabbit polyclonal) and syntaxin (rabbit polyclonal) were described previously [49]. NF200 mouse polyclonal antibody was a kind gift from Dr. M. Nassar (University of Sheffield, Sheffield, UK).

### 4.12. Statistical Analysis

All statistical analyses were performed in Graphpad Prism. Details of each individual statistical test are provided in the figure legends.

## Data Availability

The original contributions presented in this study are included in the article/Supplementary Material. Further inquiries can be directed to the corresponding author.

## Supplementary Materials

The following supporting information can be downloaded at: https://www.mdpi.com/article/10.3390/toxins18040174/s1. Figure S1: Construction of Linker 1-AB5. Figure S2: Purification of Linker 1-AB5. Figure S3: Representative immunohistochemistry images of cleaved SNAP25 (red) in the L3-L5 DRGs of ChoBot-injected naïve rats (200 ng). Table S1: Raw band densitometry analysis data for western immunoblots shown in Figure 4 and Figure 5.

## Abbreviations

The following abbreviations are used in this manuscript:

BiTox/A	Binary Toxin
BoNT(s)	Botulinum Neurotoxin(s)
BoNT/A	Botulinum Neurotoxin type A
BSA	Bovine serum albumin
CGRP	Calcitonin Gene Related Peptide
ChoBot	Cholera–botulinum toxin chimaera
CIPN	Chemotherapy-induced Peripheral Neuropathy
CMAP	Compound muscle action potential
cSNAP25	Cleaved SNAP25
CTA2	Cholera Toxin A2 subunit
CTB	Cholera Toxin B subunit
DAPI	4′,6-diamidino-2-phenylindole
Derm-Bot	Dermorphin-botulinum chimaera
DMEM/F12	Dulbecco’s Modified Eagle Medium/Nutrient Mixture F-12
DRG	Dorsal Root Ganglion
DSMZ	German Collection of Microorganisms and Cell Cultures (Deutsche Sammlung von Mikroorganismen und Zellkulturen)
EDTA	Ethylenediaminetetraacetic acid
ELISA	Enzyme-linked immunosorbent assay
el-iBoNT	Elongated isopeptide-bonded botulinum neurotoxin
FBS	Foetal bovine serum
FDA	Food and Drug Administration
FITC	Fluorescein isothiocyanate
HBTS	HEPES-buffered Tyrode’s solution
LcTd	Light chain Translocation domain
MBP	Maltose Binding Protein
Ni-NTA	Nickel–nitrilotriacetic acid
OCT	Optimal cutting temperature compound
OG	Octylglycoside
PBS	Phosphate-buffered saline
PDB	Protein Data Bank
PFA	Paraformaldehyde
RPMI	Roswell Park Memorial Institute medium
SDS	Sodium dodecyl sulfate
SDS-PAGE	Sodium dodecyl sulfate–polyacrylamide gel electrophoresis
SNAP25	Synaptosomal-associated protein 25
SNARE	Soluble N-ethylmaleimide-sensitive factor attachment protein receptor
SP-Bot	Substance P-botulinum chimaera
Syb	Synaptobrevin
TetBot	Tetanus-botulinum chimaera
TRPA1	Transient receptor potential ankyrin 1
TRPV1	Transient receptor potential vanilloid 1
