# Supplementary material for: Cholera Toxin-Mediated Targeting of Botulinum Neurotoxin Activity to Pain-Associated Sensory Neurons
Source: Toxins (Basel). 2026 Apr 3;18(4):174. doi: 10.3390/toxins18040174 (PMC13119542; doi:10.3390/toxins18040174)
Supplement: Supplementary file 1 [file toxins-18-00174-s001.zip › toxins-4133645-supplementary-english.pdf]

a) Gene sequence:

atgaaaataaaaacaggtgcacgcatcctcgcattatccgcattaacgacgatgatgttttccgcctcggctctcgccaaaat
cgaagaaggtaaactggtaatctggattaacggcgataaaggctataacggtctcgctgaagtcggtaagaaattcgagaaag
ataccggaattaaagtcaccgttgagcatccggataaactggaagagaaattcccacaggttgcggcaactggcgatggccct
gacattatcttctgggcacacgaccgctttggtggctacgctcaatctggcctgttggctgaaatcaccccggacaaagcgtt
ccaggacaagctgtatccgtttacctgggatgccgtacgttacaacggcaagctgattgcttacccgatcgctgttgaagcgt
tatcgctgatttataacaaagatctgctgccgaacccgccaaaaacctgggaagagatcccggcgctggataaagaactgaaa
gcgaaaggtaagagcgcgctgatgttcaacctgcaagaaccgtacttcacctggccgctgattgctgctgacggggttatgc
gttcaagtatgaaaacggcaagtacgacattaaagacgtgggcgtggataacgctggcgcgaaagcgggtctgaccttcctgg
ttgacctgattaaaaacaaacacatgaatgcagacaccgattactccatcgcagaagctgcctttaataaaggcgaaacagcg
atgaccatcaacggcccgtgggcatggtccaacatcgacaccagcaaagtgaattatggtgtaacggtactgccgaccttcaa
gggtcaaccatccaaaccgttcgttggcgtgctgagcgcaggtattaacgccgccagtccgaacaaagagctggcaaaagagt
tcctcgaaaactatctgctgactgatgaaggtctggaagcggttaataaagacaaaccgctgggtgccgtagcgctgaagtct
tacgaggaagagttggtgaaagatccgcgtattgccgccactatggaaaacgcccagaaaggtgaaatcatgccgaacatccc
gcagatgtccgctttctggtatgccgtgcgtactgcggtgatcaacgccgccagcggtcgtcagactgtcgatgaagccctga
aagacgcgcagactaattcgagctcgaacaacaacaacaataacaataacaacaacctcgggatcgagggaaggatttcacat
atgtccatgggcggccgcgatatcgtcgacggatccgaaaacctgtactttcagagcgcgaccgcggcgaccgtgccgccggc
ggcgccggcgggtgaaggtggtccgccggcgccgccgccgaacctgaccagcaaccgccgcctgcagcagacccaggcgcagg
tggatgaagtggtggatattatgcgcgtgaacgtggataaagtgctggaacgcgatcagaaactgagcgaactggatgatcgc
gcggatgcgctgcaggcgggcgcgagccagtttgaaaccagcgcggcgaaactgggtggcaacaacaactcctctaaggtgaa
gcgtcagatattttcaggctatcaatctgatattgatacccacaaccgtatcaaggatgaattatgacctcgaggtgaattca
cgagcaattgaccaacaaggaccatagattatgagctttaagaaaattatcaaggcatttgttatcatggctgctttggtatc
tgttcaggcgcatgcaggcggtggcacccctcaaaatattactgatttgtgcgcagaataccacaacacacaaatatatacgc
taaatgataagatcttttcgtatacagaatcgctagcgggaaaaagagagatggctatcattactttaagaatggtgcaatt
tttcaagtagaggtaccaggtagtcaacatatagattcacaaaaaaaagcgattgaaaggatgaaggataccctgaggattgc
atatcttactgaagctaaagtcgaaaagttatgtgtatggaataataaaacgcctcatgcgatcgccgcaattagtatggcaa
actaa

b) Co-expressed sequences:

MKIKTGARILALSALTTMMFSASALAKIEEGKLVIWINGDKGYNGLAEVGKKFEKDTGIKVTVEHPDKLEEKFPQVAATGDGP
DIIFWAHDRFGGYAQSGLLAEITPDKAFQDKLYPFTWDAVRYNGKLIAYPIAVEALSLIYNKDLLPNPPKTWEEIPALDKELK
AKGKSALMFNLQEPYFTWPLIAADGGYAFKYENGKYDIKDVGVDNAGAKAGLTFLVDLIKNKHMNADTDYSIAEAAFNKGETA
MTINGPWAWSNIDTSKVNYGVTVLPTFKGQPSKPFVGVLSAGINAASPNKELAKEFLENYLLTDEGLEAVNKDKPLGAVALKS
YEEELVKDPRIAATMENAQKGEIMPNIPQMSAFWYAVRTAVINAASGRQTVDEALKDAQTNSSSNNNNNNNNNLGIEGRISH
MSMGGRDIVDGSENLYFQSATAATVPPAAPAGEGGPPAPPPNLTSNRRLQQTQAQVDEVVDIMRVNVDKVLERDQKLSELDDR
ADALQAGASQFETSAAKLGGNNNSSKVKRQIFSGYQSDIDTHNRIKDEL

MSFKKIIKAFVIMAALVSVQAHAGGGTPQNITDLCAEYHNTQIYTLNDKIFSYTESLAGKREMAIITFKNGAIFQVEVPGSQH
IDSQKKAIERMKDTLRIAYLTEAKVEKLCVWNNKTPHAIAAISMAN

c) Final protein sequences in AB5-Linker complex:

SATAATVPPAAPAGEGGPPAPPPNLTSNRRLQQTQAQVDEVVDIMRVNVDKVLERDQKLSELDDRADALQAGASQFETSAAKL
GGNNNSSKVKRQIFSGYQSDIDTHNRIKDEL

GGGTPQNITDLCAEYHNTQIYTLNDKIFSYTESLAGKREMAIITFKNGAIFQVEVPGSQHIDSQKKAIERMKDTLRIAYLTEA
KVEKLCVWNNKTPHAIAAISMAN

d) Key:

Maltose binding protein
Other spacer sequence
Cholera toxin A2 (CTA2)

Periplasmic targeting sequences
Tobacco etch virus protease recognition sequence
Rattus Norvegicus synaptobrevin (residues 2-84)
Cholera toxin B with N-terminal GGG sequence (GGG-CTB)

Figure S1. Construction of Linker 1-AB5. a) Gene sequence for bicistronic construct to express b) the MBP-Linker 1 synaptobrevin-CTA2 and GGG-CTB proteins with periplasmic targeting sequences; c) the mature protein sequences following TEV-cleavage and purification as described in the methods section; d) colour coding used to highlight significant sequences in panels a), b) and c).

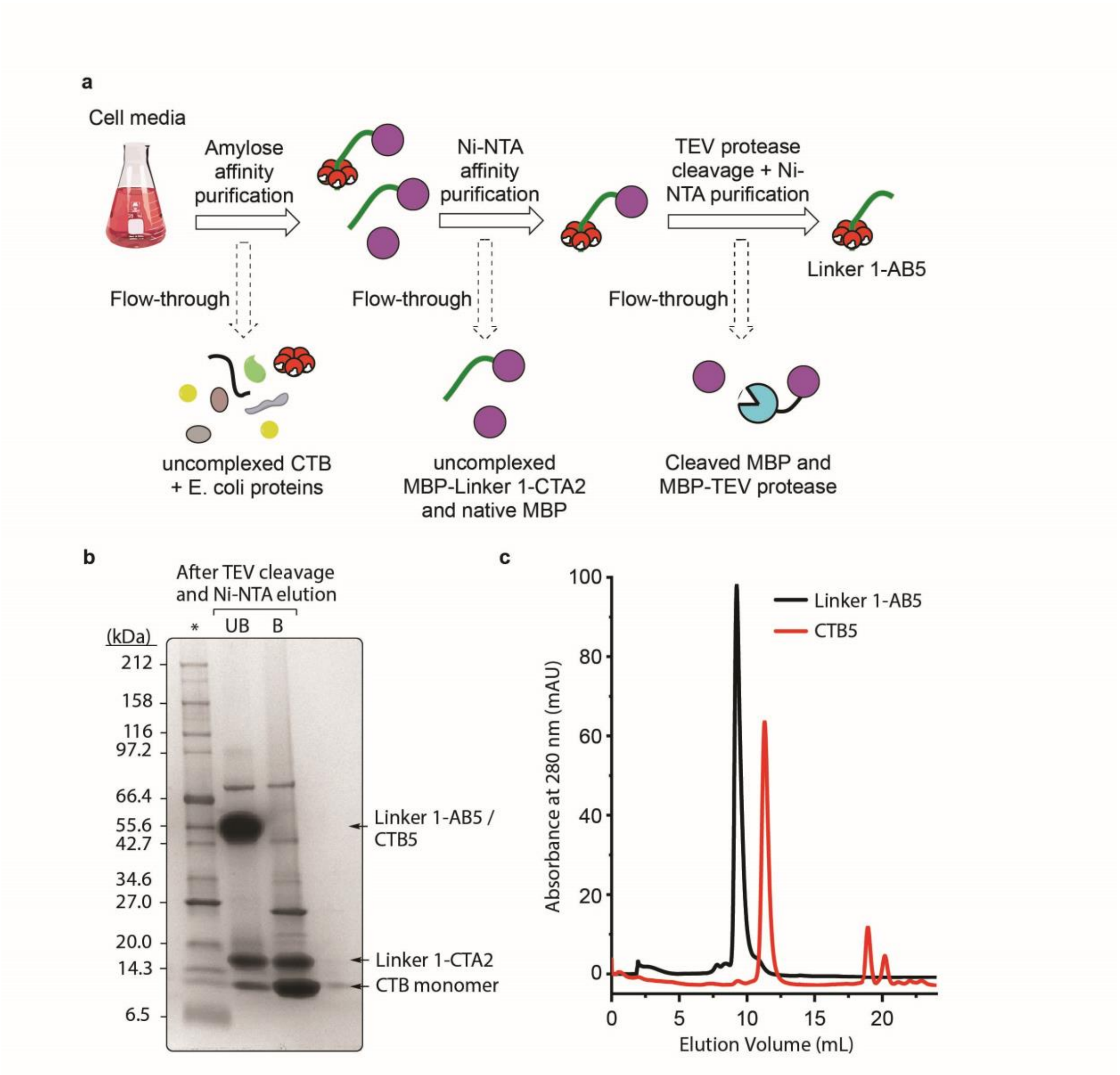

Figure S2. Purification of Linker 1-AB5. a) Schematic showing the purification strategy. Cell media is applied directly to an amylose column, isolating the MBP-tagged complex, uncomplexed MBP-Linker 1-CTA2 and native MBP. Uncomplexed CTB does not bind and is discarded with other unwanted proteins. The eluate from the amylose column is applied to a Ni-NTA affinity column, resulting in purification of MBP-Linker 1-AB5 and loss of uncomplexed MBP-Linker 1-CTA2 as well as native MBP. The purified protein is treated with MBP-tagged TEV protease, resulting in cleavage of the MBP tag from Linker 1-AB5. Purification of Linker 1-AB5 is achieved through a second round of Ni-NTA affinity chromatography, resulting in loss of MBP and TEV protease, and purification of Linker 1-AB5. b) SDS-PAGE (4-15% acrylamide) analysis of Ni-NTA chromatography elution following cleavage with MBP-TEV. In the unboiled sample, a thick band at ~55 kDa can be observed to disappear upon boiling, which is likely to be the CTB5 pentamer. A band at ~15 kDa seen in both lanes corresponds to Linker 1-CTA2. c) Chromatogram of gel filtration chromatography of Linker 1-AB5 complex (black) using a Superdex 75 Increase 10/300 GL gel filtration column, overlaid with a trace of purified CTB5 (red), showing retention volume (mL) against

absorbance at 280 nm (mAU). A single peak was observed for Linker 1-AB5 that eluted with a retention volume of 9 mL. In contrast, CTB5 elutes with a retention volume of ca. 12 mL. The lower retention volume of Linker 1-AB5 is consistent with having a higher hydrodynamic radius than the CTB5 pentamer.

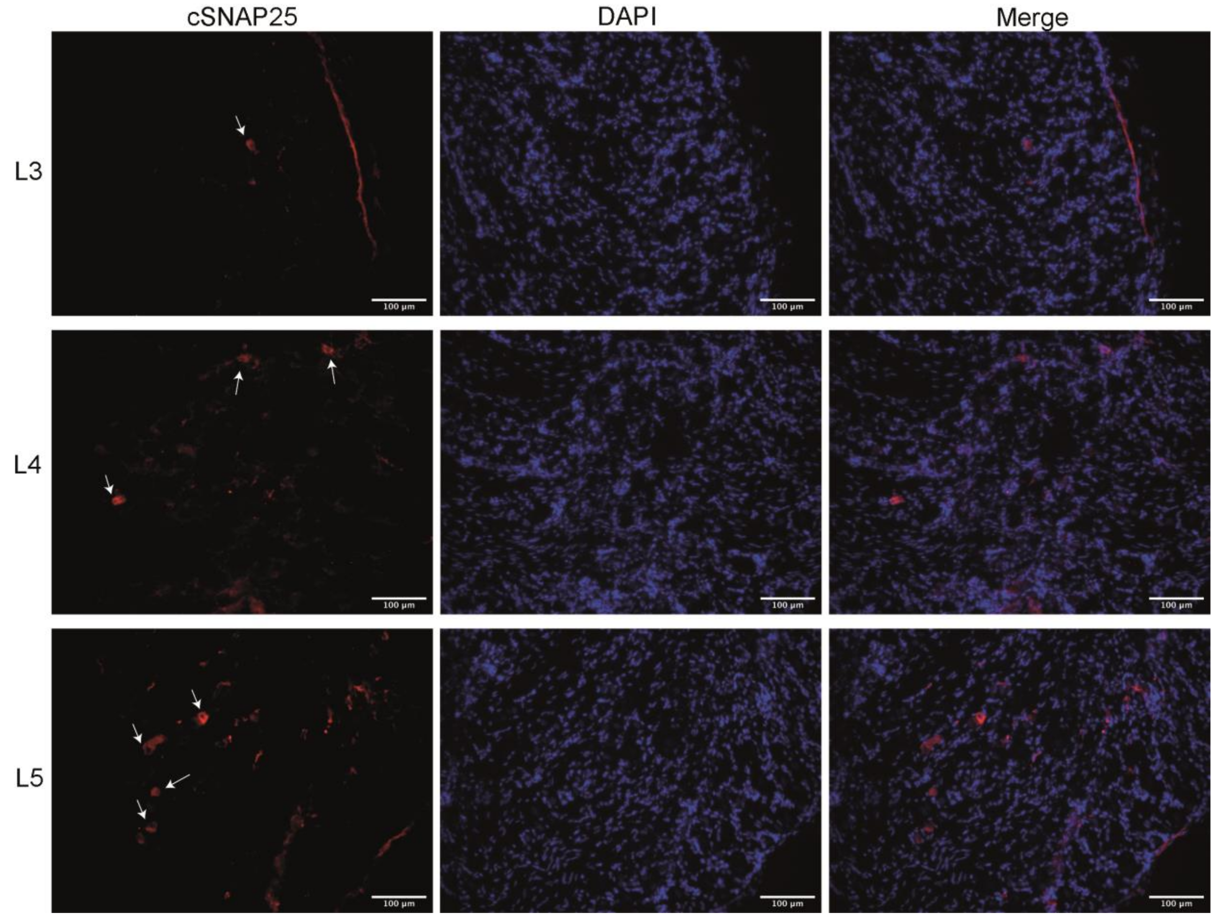

Figure S3. Representative immunohistochemistry images of cleaved SNAP25 (red) in the L3-L5 DRGs of ChoBot-injected naïve rats (200 ng). Arrows denote cleaved SNAP25-immunoreactive soma. The L5 DRG exhibits the highest number of cells with cleaved SNAP25.

Condition	Syntaxin	SNAP25	Cleaved SNAP25	Total SNAP25	%Cleaved SNAP
DRG 0 pM	60.932	81.56	0	81.56	0
DRG 400 pM LcTd	21.643	90.621	7.073	97.694	7.239953
DRG 0.64pM ChoBot	44.716	79.75	63.413	143.163	44.29427
DRG 3.2 pM ChoBot	46.603	49.567	61.304	110.871	55.29309
DRG 16pM ChoBot	47.788	67.5	127.153	194.653	65.32291
DRG 80 pM ChoBot	47.893	82.698	150.78	233.478	64.57996
DRG 400 pM ChoBot	50.885	87.669	145.635	233.304	62.42285
SiMa cells ChoBot 0pM	8.33	72.52	0	72.52	0
SiMa cells ChoBot 0.025 pM	7.69	67.89	0	67.89	0
SiMa cells ChoBot 0.12 pM	8.961	70.88	21.437	92.317	23.22108
SiMa cells ChoBot 0.64 pM	13.319	70.558	39.475	110.033	35.8756
SiMa cells ChoBot 3.2 pM	9.435	45.783	65.174	110.957	58.73807
SiMa cells ChoBot 16 pM	11.66	15.655	68.615	84.27	81.42281
SiMa cells ChoBot 80 pM	8.69	16.364	74.186	90.55	81.92822
SiMa cells ChoBot 400 pM	6.305	8.52	64.451	72.971	88.32413
SiMa cells ChoBot 2000 pM	7.058	0	50.172	50.172	100
SiMa cells ChoBot 10000 pM	6.451	0	48.73	48.73	100
SiMa cells LcTd 16 pM	17.853	61.982	0	61.982	0
SiMa cells LcTd 80 pM	20.982	70.762	0	70.762	0
SiMa cells LcTd 400 pM	23.136	72.423	0	72.423	0
SiMa cells LcTd 2000 pM	19.305	57.183	10.204	67.387	15.14239
SiMa cells LcTd 10000 pM	13.192	42.312	28.142	70.454	39.94379
ChoBot timecourse 0h	20.233	95.314	0	95.314	0
ChoBot timecourse 4h	10.211	19.192	42.424	61.616	68.85225
ChoBot timecourse 6h	17.312	26.255	67.355	93.61	71.95278
ChoBot timecourse 8h	25.369	25.172	63.452	88.624	71.59686
ChoBot timecourse 24h	15.17	19.452	112.075	131.527	85.21064
ChoBot timecourse 48h	9.377	13.783	92.91	106.693	87.08163
ChoBot timecourse 72h	5.987	13.419	94.568	107.987	87.5735

Table S1. Raw band densitometry analysis data for western immunoblots shown in Figures 4 and 5.
